# Navigating Now and Next: Recent Advances and Future Horizons in Robotic Radical Prostatectomy

**DOI:** 10.3390/jcm13020359

**Published:** 2024-01-09

**Authors:** Abrar H. Mian, Matthew K. Tollefson, Paras Shah, Vidit Sharma, Ahmed Mian, R. Houston Thompson, Stephen A. Boorjian, Igor Frank, Abhinav Khanna

**Affiliations:** 1 Department of Urology, Mayo Clinic, Rochester, MN 55905, USA; 2 Urology Associates of Green Bay, Green Bay, WI 54301, USA

**Keywords:** robotic-assisted radical prostatectomy, Single Port, pelvic fascia sparing, artificial intelligence, continence

## Abstract

Robotic-assisted radical prostatectomy (RARP) has become the leading approach for radical prostatectomy driven by innovations aimed at improving functional and oncological outcomes. The initial advancement in this field was transperitoneal multiport robotics, which has since undergone numerous technical modifications. These enhancements include the development of extraperitoneal, transperineal, and transvesical approaches to radical prostatectomy, greatly facilitated by the advent of the Single Port (SP) robot. This review offers a comprehensive analysis of these evolving techniques and their impact on RARP. Additionally, we explore the transformative role of artificial intelligence (AI) in digitizing robotic prostatectomy. AI advancements, particularly in automated surgical video analysis using computer vision technology, are unprecedented in their scope. These developments hold the potential to revolutionize surgeon feedback and assessment and transform surgical documentation, and they could lay the groundwork for real-time AI decision support during surgical procedures in the future. Furthermore, we discuss future robotic platforms and their potential to further enhance the field of RARP. Overall, the field of minimally invasive radical prostatectomy for prostate cancer has been an incubator of innovation over the last two decades. This review focuses on some recent developments in robotic prostatectomy, provides an overview of the next frontier in AI innovation during prostate cancer surgery, and highlights novel robotic platforms that may play an increasing role in prostate cancer surgery in the future.

## 1. Introduction

Radical prostatectomy has experienced a wave of innovation over the past century. Initially described by Hugh Hampton Young using the perineal approach in 1905 [1], the procedure has undergone significant advancements leading to greatly improved oncologic and functional outcomes over the last century. With the inception of Millin’s retropubic approach in 1948 and the subsequent introduction of PSA and transrectal ultrasound several decades later, there were marked reductions in morbidity and mortality due to refinements in surgical techniques and early cancer detection [2,3,4]. While a vast majority of the history of radical prostatectomy chronicles the open surgical approach, recent years have seen a shift towards minimally invasive techniques.

Robotic-assisted radical prostatectomy (RARP), the latest development in this lineage, has been both promising and a subject of debate. Introduced in 1998, the first robotic platform revolutionized the field, equipped with a 3D camera for depth perception and wristed instruments mimicking the dexterity of human hands [5]. By 2004, Menon et al. began to document their pioneering technique for RARP [6]. This method dramatically altered the surgical landscape for prostate cancer: recovery periods were reduced from weeks to days, and the need for blood transfusions substantially decreased [7]. However, these promising results did not come without controversy. Despite the lack of prospective randomized clinical trials proving its superiority over open prostatectomy, its ensuing popularity among hospitals, physicians, and patients drove its widespread adoption. The high cost of surgical robots, ranging between USD 3 and USD 4 million, led to disparities in access to this technology across sociodemographic regions [8,9]. Furthermore, recent studies have suggested that the primary determinant of outcomes is not the surgical tool used, but rather the volume of cases undertaken by a center, with higher-volume centers consistently demonstrating more favorable surgical outcomes [10,11,12,13,14,15]. Nonetheless, RARP has been embraced as the gold standard approach to radical prostatectomy. In fact, 85% of all radical prostatectomies were performed robotically by 2013 [16]. Retrospective data suggest that RARP offers comparable oncologic and functional outcomes to open surgery, and its minimally invasive nature reduces blood transfusion, shortens postoperative recovery times, and may enhance patient quality of life [17].

With its relatively recent inception, RARP has continually evolved. This review seeks to spotlight these recent technical innovations, assess the current state of RARP, and explore promising new technologies in robotic surgery.

## 2. Single Port Surgery

The da Vinci robotic platform (Intuitive Surgical, Sunnyvale, CA, USA) is the most commonly used modem in the field of robotic surgery [18]. For years, it employed a multi-arm design, which was incrementally improved over several iterative editions of the da Vinci robot. In an attempt to minimize the number of incisions and further reduce surgical morbidity, surgeons began to attempt single-site radical prostatectomies with the multiport robot, often achieved with all robotic trochars placed through a single laparoscopic gel-port [19]. However, this posed several challenges, such as clashing of robotic instruments due to inadequate working distance between ports, lack of triangulation, and limitations during fine movements, such as suturing. In 2010, White et al. postulated that their single-site RARP using the multi-arm da Vinci robot reduced instrument crossing, improved ergonomics, and facilitated instrument tip articulation [19]. However, challenges with this technique persisted, and single-site RARP with the multi-arm robotic platform achieved only very limited adoption.

In 2018, the Food and Drug Administration approved the da Vinci Single Port (SP) system for urologic surgery, ushering in a fresh, new wave of surgical innovation in RARP. The new device had capabilities similar to its multiport predecessor; however, three articulating endoscopic instruments and a camera were now inserted coaxially through a single laparoscopic trochar [20]. Since its inception, several surgical centers have reported their experiences. Herein, we will review the transperitoneal, extraperitoneal, perineal, and transvesical approaches to RARP, many of which have been facilitated by the arrival of the SP platform.

### 2.1. Transperitoneal Approach

The transperitoneal approach was the initial method utilized for RARP with the novel SP platform, aiming to replicate well-established transperitoneal approaches from the multi-arm platform [21,22]. Kaouk et al. presented their initial results from a cohort of 46 patients who underwent this approach. The median length of the postoperative hospital stay was 25.7 h, with 44/46 (95.6%) of patients requiring opioids. These patients had a 90-day continence rate of 62.5%, positive surgical margins (PSMs) in 41.3%, and a 90-day undetectable PSA rate of 84.2% [23]. As surgical experience with the SP platform grew, surgical morbidity and oncologic outcomes continued to improve. In a subsequent, larger cohort of 238 patients, 138 cases were successfully performed without the need for a separate assistant port site. The median postoperative hospital stay was 14 h, and the PSM rate was 26.9%. Postoperative continence was reported as 40.2%, 63%, and 87.2% at 6 weeks, 3 months, and 6 months, respectively [24].

Vigneswaran conducted a retrospective analysis of 45 patients undergoing SP RARP. Of these, 30% were pain-free on the first postoperative day, and the median hospital length of stay was one day. They observed high PSMs at 42%, which they attributed to the prevalent use of nerve sparing and the high rate of locally advanced disease. Furthermore, 86% of patients exhibited recovery of urinary continence 90 days post-surgery [25].

Moschovas et al. compared the SP transperitoneal approach to the multiport method, with 71 patients in each group. While they did not specify the postoperative length of stay, they highlighted that no statistically significant differences existed between the groups. In the Single Port SP cohort, positive surgical margins were observed in 17% of cases, with no cases of biochemical recurrence (BR) in a median follow-up of 4.4 months. Postoperative continence was achieved in 4.2%, 48%, and 68% at 7, 45, and 90 days, respectively [26].

Comparative results of multi-arm versus SP RARP during the early period of SP adoption should be taken in context. Firstly, the relative novelty of the SP port approach implies that these early cohorts were effectively comparing surgeons with mature multiport experience to their early learning curve with SP. Moschovas et al. emphasized the value of using an assistant port during this learning phase to avoid the unnecessary use of excessive electrocautery [26]. Other challenges, such as a limited range of motion and a lack of sweeping power, were also identified in the SP approach [27]. Nonetheless, despite these challenges, early comparative studies suggested superior postoperative pain and hospital length of stay with SP, without significantly compromising other perioperative and oncologic outcomes [25]. As techniques and experience with SP evolved, outcomes continued to further improve.

### 2.2. Extraperitoneal Approach

Following the early experience with transperitoneal SP RARP, the next adaptation in this realm was the extraperitoneal approach. This method appeared to address some of the limitations associated with the transperitoneal approach. Most notably, it bypasses the peritoneal cavity, allowing for safe and technically reproducible results even among patients with complex prior surgical histories [28]. The extraperitoneal approach also brings with it other benefits, such as minimal peritoneal irritation from insufflation, reduced bowel manipulation, and lesser requirements for steep Trendelenburg positioning [29]. However, it is worth noting that for patients who have previously undergone procedures like inguinal hernia repair or kidney transplantation, there could be substantial scarring in the extraperitoneal space, which might complicate this surgical approach [30].

Kim et al. shared their short-term outcomes from a cohort of 157 patients who underwent extraperitoneal SP RARP, highlighting that 70% of these patients were discharged the same day. They observed PSMs in 29% of cases and a BR rate of 8.3% at 9-month follow-up. Additionally, 82.5% were continent and 64.4% achieved potency 9 months post-surgery [31]. Wilson et al. presented their findings from 60 patients treated with this approach, revealing a median postoperative stay of 4.2 h, 23% incidence of PSMs, and 76% of patients achieving continence at the 90-day mark [32]. Similarly, Kaouk et al. discussed their experience with the first 10 patients treated with this approach: the median postoperative stay was recorded as 0 days, PSMs were identified in 50% of the participants, and 50% were continent by the 90th day post-surgery [33].

Zeinab et al. compared the extraperitoneal and transperitoneal approaches retrospectively, with each cohort consisting of 238 patients. Patients in the extraperitoneal cohort had a significantly greater history of prior abdominal surgeries. While the extraperitoneal cohort had a longer operative time and higher estimated blood loss, it also enjoyed a significantly shorter postoperative length of stay (7.5 h vs. 14 h in extraperitoneal versus transperitoneal, respectively). No significant differences were noted in intraoperative complications, PSMs, or postoperative continence [24].

Collectively, these studies established the safety and efficacy of extraperitoneal approaches to RARP. Across most studies, surgeons acknowledge a pronounced learning curve, particularly during the initial early adoption period of this approach. However, outcomes rapidly improved as familiarity and experience with the technique grew. Despite being a relatively recent innovation, the extraperitoneal approach has already showcased advantages over traditional transperitoneal RARP, such as reduced hospital length of stay, while maintaining functional and oncologic outcomes comparable to the transperitoneal method.

### 2.3. Perineal Approach

The perineal approach to radical prostatectomy was first described by Young in 1905 [1]. However, the subsequent description of open retropubic radical prostatectomy largely replaced perineal approaches. The advent of robotic surgery generally replicated open retropubic approaches, as attempts at robotic perineal RARP were often limited by a tight working space and instrument clashing. With the development of the SP robot, however, many of the shortcomings of perineal robotic RARP could theoretically be addressed. Robotics enhances both the visual clarity and surgical ergonomics of the perineal approach [34]. Moreover, it offers an opportunity to avoid the peritoneal space, which may be densely scarred in patients with a history of complex prior abdominal surgeries.

Lenfant et al. studied their experience with a cohort of 26 patients, reporting a median hospital stay of 23 h. A notable contrast was observed in the reduced need for postoperative pain medications compared with patients who had undergone multiport transperitoneal surgery. A high PSM rate of 65.4% was reported, hypothesized to stem from the narrow surgical field, which hindered the introduction of a specimen retrieval bag. Interestingly, only one patient showed signs of BR at the one-year mark. Furthermore, by the third month post-surgery, 75% of the patients had regained continence [35].

Yu et al. reported on their cohort of 50 patients undergoing perineal RARP. They observed PSMs in 10% of cases and just one instance of BR at the one-year follow-up. Continence evaluations, stratified temporally by their first 20 and subsequent 30 patients, revealed return of urinary continence rates of 32%, 42%, 79%, and 95% for the former group, and 48%, 80%, 96%, and 100% for the latter—assessed at 1, 3, 6, and 12 months postoperatively, respectively [36]. These findings underscore the learning curve associated with this new approach, as surgical outcomes during the initial experience may not reflect outcomes once the technique is mature.

Chang et al. outlined outcomes for their six-patient cohort, recording a median hospital stay of 2 days, PSMs in 33%, and, by the end of the third postoperative month, 83% of patients had achieved continence.

The nascent status of this approach and the paucity of prospective investigations and randomized clinical trials make broad generalizations challenging. Nonetheless, these small, initial studies suggest the safety and feasibility of perineal RARP, an innovative approach reserved for only the most challenging situations in which extensive prior abdominal surgery precludes retropubic approaches.

### 2.4. Transvesical Approach

Another approach leveraging the SP robot’s capability in tight anatomical spaces is the transvesical approach. Initially described by Sawczyn et al., this approach provides extraperitoneal access, avoids potential adhesions from prior abdominal surgery, and offers maximal preservation of the Space of Retzius and also the Rectovesical Pouch [37].

Zeinab et al. studied 78 patients who underwent transvesical SP RARP, reporting a median hospital stay of 5.5 h. PSMs were observed in 15% of patients, with one instance of BR at six months. Postoperatively, continence rates were 72% at 6 weeks, 97% at 3 months, and 100% at 6 months. Regarding potency, 52% of patients had a preoperative Sexual Health Inventory for Men (SHIM) score ≥ 17, which decreased by 28.6% at six months post-operation [38]. A subsequent study of 100 patients who underwent transvesical SP RARP by the same surgeon confirmed a similar postoperative length of stay and PSM rates, suggesting the safety and efficacy of this novel approach [39]. Deng et al.’s retrospective cohort of 60 patients revealed PSMs in 15%, with 3 patients experiencing BR. A total of 90% of patients achieved continence immediately after catheter removal, with 100% achieving continence by 3 months. The median preoperative International Index of Erectile Function—5 (IIEF-5) score was 17, which decreased to 13 at 3 months post-surgery [40]. Zhou et al. examined 72 patients, in whom PSMs were noted in 20.8%. There was no recorded BR at six months following surgery. All patients achieved continence within two weeks post-surgery. IIEF-5 scores were 11 preoperatively and remained stable at 12 at the three-month follow-up [41].

These relatively small retrospective studies establish the safety and feasibility of the transvesical approach to RARP. While the lack of long-term follow-up precludes a comprehensive analysis of longitudinal oncologic outcomes, early results appear to imply the oncologic safety of this approach. Early return of continence outcomes in these initial studies are promising, and this approach warrants further evaluation in larger, prospective studies.

### 2.5. SP Limitations

As with any new surgical technology, there are limitations of the SP robot that should be taken into account. The SP robot excels in tight working spaces in which there may not be enough working room to accommodate multi-arm robotic platforms. These include transvesical, preperitoneal (i.e., extraperitoneal), and transperineal approaches. However, the SP robot may be at a slight disadvantage compared to the multiport robot for the transperitoneal approach, because the SP is limited to work within a confined working space, and transperitoneal surgery requires broad instrument movements to optimize tissue traction and blunt dissection within the peritoneal cavity. Thus, SP may be best suited to extraperitoneal approaches, whereas traditional multiport robotic platforms may excel inside the peritoneal cavity. Also, the SP robot arms may not have the same tensile strength as the multiport robot, and thus for very large glands or very large median lobes in which instrument strength is required, the SP may be less optimal.

## 3. Pelvic-Fascia-Sparing Techniques

Radical prostatectomy is highly effective for the treatment of localized prostate cancer [16]. However, this surgery does have notable impacts on patient quality of life, including the risk for both short- and long-term urinary incontinence [42]. In an endeavor to mitigate the impact of surgery on urinary function, numerous technical modifications to RARP have been introduced. These largely focus on preserving or restoring key pelvic anatomical structures that play a pivotal role in continence [43,44,45,46]. Techniques that preserve the natural anatomy, such as bladder neck preservation and urethral length preservation, show promise in uniformly improving short-term urinary continence. Reconstructive techniques, such as bladder neck reconstruction, reapproximation of Denonvillier’s fascia to periurethral tissues (posterior reconstruction), and anterior urethral suspension, have demonstrated somewhat inconsistent results [43,46,47,48,49,50,51,52,53,54,55,56,57,58]. In 2010, Galfano et al. introduced a technique that focused on preserving the natural pelvic anatomy by sparing the space of Retzius [59]. The posterior approach to the prostate preserves key anatomical structures implicated in urinary continence, such as the dorsal vein complex, the puboprostatic ligaments, the detrusor apron, and the striated sphincter [60].

### 3.1. Retzius Sparing

Several prospective and randomized trials have evaluated the Retzius-sparing approach. The current review primarily focuses on postoperative urinary continence and surgical margins. While the former generally showcases quite promising outcomes with this technique, the latter remains under scrutiny. A limited number of studies have also touched upon the preservation of sexual potency, albeit using heterogeneous endpoints. Umari et al. presented a prospective series involving 282 patients who underwent Retzius-sparing RARP. They reported an immediate postoperative continence rate of 70.4% and a PSM rate of 15.6%. The team employed the IIEF-5 to evaluate sexual potency, noting median scores of 7.61, 7.83, 8.08, 9.03, and 8.90 at 1, 3, 6, 9, and 12 months, respectively [61]. Egan et al. detailed another prospective cohort comprising 70 patients. Their findings included a twelve-month continence rate of 95%, a PSM rate of 34.3%, and a 65.7% rate of sexual potency one year after surgery [62]. In the Retzius-sparing arm of a randomized control trial, Menon et al. observed an immediate continence rate of 76%, improving to 96% six months later. The study also recorded a 25% PSM rate, with 86.5% of participants achieving erections sufficient for sexual intercourse 12 months after the operation. Comparatively, in the anterior RARP control arm, 60% of patients achieved continence at 3 months, 74% at 6 months, and 88% at 9 months. PSMs were seen in 13% of patients, and 69.2% had erections sufficient for sexual intercourse at 12 months [63]. Several other studies corroborate the efficacy of Retzius sparing in mitigating the impact of RARP on urinary function, reporting continence rates ranging between 69% and 100% over various postoperative periods and PSM rates spanning 23.3% to 28.2% [64,65,66,67,68,69]. These studies all report quite dramatic improvements in early urinary control following Retzius-sparing RARP, implying the integral role of anterior anatomic support of the bladder and urethra in postoperative continence.

A recent meta-analysis that compared the Retzius-sparing technique to the standard anterior approach in RARP revealed a notably higher immediate continence rate with the Retzius-sparing approach (RR = 1.81; 95% CI = [1.26–2.60]). Continence rates at 3 and 6 months were also higher for the Retzius-sparing technique (RR = 1.57; 95% CI = [0.69–3.58] and RR = 1.22; 95% CI = [0.89–1.66], respectively), though no discernible difference was appreciated at 12 months (RR = 1.14; 95% CI = [0.98–1.32]). Reinforcing the concern of elevated PSM rates with this approach, the meta-analysis identified a statistically significant increase in PSM rates for ≤pT2 tumors following the Retzius-sparing approach ((RR) = 1.39; 95% (CI) = [1.01–1.91]). Collectively, these findings highlight the potential benefits of the Retzius-sparing technique in enhancing early postoperative urinary continence, yet the increased PSM rate is an area of ongoing concern. Although early urinary outcomes appear promising, the lack of long-term follow-up in most studies precludes a comprehensive analysis of the durable oncologic efficacy of this approach [70].

### 3.2. Hood Sparing

In hopes of replicating the anatomic preservation of anterior pelvic structures seen in Retzius-sparing approaches while also aiming to mitigate the potentially unfavorable PSM rates seen with Retzius sparing, Wagaskar et al. reported their experience with the “Hood-sparing technique”. In their approach, the preserved anterior tissue following prostate removal (including detrusor apron, endopelvic fascia, and puboprostatic ligaments) has a “hood” appearance, which is thought to provide support to the membranous urethra, external sphincter, and vesicourethral anastomosis [71]. In their series of 300 patients, 21% achieved early continence within one week of surgery, 83% in one month, and 91% at three months. Shimmura et al. also reported similar results following their experience with the Hood-sparing technique in combination with umbilical ligament preservation. In their series of 42 patients undergoing this modified technique, 35.7% saw a return to continence at 2 weeks, 69.1% at 1 month, 90.5% at 3 months, and 100% at 6 months [72]. Zhang et al. adapted the Hood-sparing technique for SP robots and also achieved similar continence outcomes. Of 24 patients, 54% saw continence recovery at 1 week, 75% at 1 month, 92% at 3 months, and 96% at 12 months [73]. Similar outcomes are seen across almost all studies regarding both early return to urinary incontinence as well as long-term continence. Interestingly, the PSM rate was similar between Wagaskar et al. and Zhang et al. at 6% and 8%, respectively, while Shimmura et al. reported a 16% PSM rate. Although there are no prospective randomized trials to compare surgical techniques, these retrospective studies show promise, as the “Hood-sparing technique” seems to offer promising urinary continence results while potentially overcoming the PSM challenges associated with Retzius sparing (Table 1).

Several explanations have been postulated for these observations. The Hood-sparing technique may offer a more accessible and replicable approach, as it largely replicates the traditional anterior approach to RARP with which many surgeons already have familiarity. These factors may play a role in the reduced incidence of PSMs observed with Hood sparing as opposed to Retzius sparing, while still preserving much of the anatomic support believed to be responsible for postoperative urinary continence [71,72,74].

In order to validate these findings and better understand the efficacy of both Hood-sparing and Retzius-sparing approaches, large-scale, prospective, randomized controlled trials are necessary. To our knowledge, there is one such trial underway. A prospective, multi-centered, randomized controlled trial termed the “PARTIAL” trial aims to compare pelvic-fascia-sparing techniques (including both Retzius and Hood sparing) against the conventional anterior approach to RARP. The results from this trial will be pivotal in providing definitive evidence regarding the impact of these novel surgical approaches.

## 4. Preserving Erectile Function

In addition to urinary control, erectile dysfunction is the other major impact of radical prostatectomy on patient quality of life. Despite Walsh and Donker’s landmark description of peri-prostatic neural anatomy in 1982 [75], achieving a consistent return of erectile function following radical prostatectomy remains challenging. Although patient factors play a critical role in postoperative sexual function, several intra-operative technical considerations are also of interest. This section focuses on a novel concept in this field: the use of dehydrated human amnion/chorion membrane (dHACM) allografts placed on the neurovascular bundles during surgery, which is postulated to accelerate the early return of sexual function. Perinatal tissue membranes are recognized sources of cytokines, growth factors, and neurotrophic factors that can mitigate the inflammatory response and improve potency outcomes [76]. dHACM contains growth factors known to stimulate epithelial cell migration and proliferation, such as PDGF-AA, PDGF-BB, TGFα, TGFβ1, bFGF, EGF, and GCSF. These are thought to work synergistically to initiate the healing process. Additionally, dHACM houses various chemokines and cytokines (IL-4, 6, 8, and 10), which may contribute to its roles in reducing inflammation, promoting angiogenesis, and enhancing wound healing [77].

Translating these principles into practice, Ogaya-Pinies et al. compared 235 patients who received bilateral placement of dHACM grafts around the neurovascular bundle during RARP with 705 who did not. The mean time to recovery of sexual function was significantly shorter in the graft group at 2.37 months compared to 3.94 months in the comparison group. Significant differences were observed in groups that underwent both full and partial nerve sparing. Importantly, the placement of dHACM allografts did not impact PSM or BR [78]. Patel et al. shared their experience with dHACM allograft placement in 58 patients. While they observed no significant difference in potency or continence outcomes, they did find a marked reduction in the mean time to achieving both, including a mean time to potency of 1.34 months and a mean time to continence of 1.21 months among patients with graft placement compared to 3.39 months and 1.83 months, respectively, in the control arm. Furthermore, at the most recent follow-up, postoperative SHIM scores were 16.2 in the allograft group versus 9.1 in the control group [79]. Razdan retrospectively analyzed a large cohort of 1400 patients undergoing RARP, 700 of whom received dHACM allografts. They concluded that dHACM allograft placement was independently associated with a higher likelihood of achieving erectile recovery at 1 year. Furthermore, patients receiving allografts during surgery were 3.86 times more likely to achieve potency at any given time point after surgery when compared to their control group [80].

Although the current data are retrospective, they hold promise for optimizing erectile function after RARP. Currently, a prospective, randomized, placebo-controlled trial is underway [81], which should provide further clarity on the role of dHACM allografts in postoperative erectile recovery after RARP.

## 5. Lymph Node Dissection

As discussed, the Single Port (SP) robot introduces innovative surgical approaches, including transvesical, preperitoneal, and transperineal methods. These approaches, while groundbreaking in their minimal invasiveness and precision, present certain limitations in accessing iliac and obturator lymph nodes, which are integral to comprehensive lymph node dissection (LND). For a more extensive LND, particularly in the case of extended pelvic lymph node dissection, the transperitoneal approach utilizing the da Vinci Xi robotic system may be the most optimal approach [82]. This approach facilitates a broader and more thorough exploration of the lymphatic territories due to better exposure to the iliac vessels and presacral area, particularly with transperitoneal approaches. Recent advancements in lymph node visualization techniques, including, notably, the application of indocyanine green (ICG), have significantly enhanced the efficacy of lymphatic dissection. The use of ICG in conjunction with near-infrared fluorescence imaging has shown promise in improving the identification and mapping of lymph nodes during surgery, as evidenced by the emerging literature [83,84,85]. This technological synergy not only augments surgical precision but also potentially improves oncological outcomes by enabling more accurate lymph node staging and removal.

## 6. Artificial Intelligence in Radical Prostatectomy

The recent surge in artificial intelligence (AI) has garnered interest across all fields of medicine and surgery. AI has recently been used in RARP to evaluate surgical performance and predict clinical outcomes. Although validated assessment tools, such as the Global Evaluative Assessment of Robotic Surgery, have proven to be predictive of certain surgical outcomes, their reliance on manual review of surgical videos introduces a subjective element and makes the process of surgeon assessment labor-intensive [86,87,88]. In 2018, Hung et al. introduced the first example of the utility of machine learning algorithms in evaluating surgical performance during RARP. By using automated performance metrics (APM) as the training input and length of stay (indicative of surgeon experience) as the outcome, they trained algorithms that accurately predicted the length of stay following RARP in 78 patients. Out of the three models assessed, the Random Forest—50 machine learning model achieved a predictive accuracy of 88.5%, giving it significant prognostic value in surgical duration, hospital length of stay, and foley catheter duration [89].

In another study, Hung et al. once again sought to utilize different machine learning algorithms to predict postoperative urinary continence in patients undergoing RARP. The authors demonstrated that a deep learning model was able to predict postoperative continence and that surgeons with more efficient APMs achieved higher continence rates at 3 and 6 months postoperatively [90].

Most recently, Schuler et al. reported their experiences with AI in predicting surgeon caseload and expertise in nerve-sparing RARP. A total of 35 urologists were categorized as high-volume or low-volume surgeons based on a case threshold of 250 nerve-sparing RARPs. These urologists completed demographic surveys, reviewed simulated case presentations, and performed simulated nerve-sparing RARP. Features for the machine learning methods were generated from robotic objective performance indicators, video-based surgical gestures, and model-based force sensors. The machine learning methods were utilized to create models to judge surgeon experience, and the models were tested to ensure reliability. A logistic regression (LR) model achieved 96% accuracy in predicting individual surgeon experience [91].

Overall, there is much potential in utilizing AI to objectively assess surgical performance and predict clinical outcomes. Additionally, AI has shown promise in automatically detecting the key steps of surgery during RARP [92]. Step detection serves as the foundation for numerous potential future applications of AI in surgery, including surgeon education and training, quality benchmarking, optimizing operating room logistics, and potentially even real-time intraoperative decision support during surgery in the future.

## 7. Novel Robotic Platforms

Lastly, this review will highlight emerging robotic platforms. To date, the vast majority of RARPs have been performed using the da Vinci platform from Intuitive Surgical. However, in recent years, several promising alternatives have emerged. The Hugo RAS (Medtronic, Minneapolis, MN, USA) robot boasts the most available literature amongst novel robotic surgical platforms. Distinct from the conventional da Vinci robot, the Hugo incorporates standalone arm carts for each robotic arm, a unique controller design, and an open-console setup featuring three-dimensional (3D) high-definition glasses. The first documented case series was reported by Bravi et al., detailing their experiences with six patients undergoing RARP with the Hugo system [93]. In 2023, the same group published a comprehensive comparative study contrasting the Hugo robot and the da Vinci, which included 164 patients in the Hugo cohort and 378 in the da Vinci cohort. Although not statistically significant, the Hugo robot exhibited a slightly extended median operative time of 180 min compared to the da Vinci’s 165 min, which likely reflects the learning curve associated with a novel surgical platform. The median duration of hospital stay was similar across both groups, with no notable variations in postoperative complications or PSMs [94]. Alfano et al. retrospectively assessed fifteen patients treated using the new platform, noting a median operative duration of 235 min and only a single postoperative complication (gastrointestinal bleed due to gastritis). The average hospital stay was two days [95]. Ragavan et al. also conducted a comparative study between the Hugo and da Vinci robots, with each robot utilized in 17 patients. The results indicated no meaningful differences in total operative and docking times, hospital stay duration, PSMs, continence, or PSA at a 3-month follow-up. The only significant difference between groups was the da Vinci robot’s superior median lymph node yield, a discrepancy the researchers speculated might stem from the higher prevalence of T3/high-risk disease patients in the da Vinci group [96]. Marques-Montiero et al. presented the inaugural series of extraperitoneal RARP performed with the Hugo platform. Their findings revealed median console and operative times of 152 and 210 min, respectively. The average hospital stay was two days, with a single postoperative complication observed within the first thirty days [97]. A recurring challenge emphasized across studies was the longer docking time due to the involvement of multiple independent carts. However, Bravi et al. opined that increased familiarity could mitigate this issue, as their team ultimately managed to achieve comparable operative times without variations in margin rates or functional outcomes when compared to the da Vinci robot [94].

The Revo-i^®^ robotic platform (Meere Company Inc.) is another new robot that has shown promise for RARP. Comprising a four-arm robotic operation cart, a surgeon control console, a high-definition vision cart, and reusable endoscopic tools, the platform’s first documented use for RARP was presented by Chang et al. in 2018. Their findings showed median console and operative durations of 92 and 182 min, respectively. Four patients experienced PSM, with one single instance of BR 3 months after surgery [98]. A study by Alip et al. compared the Revo-i platform with the da Vinci, including 33 patients for each robotic system. The da Vinci robot displayed significantly reduced console, operative, and suturing durations, whereas the Revo-i robot led to a notably shorter hospitalization period. Notably, all surgeries were performed by a single, highly experienced robotic surgeon. While the authors acknowledged a learning curve associated with the new system, they emphasized the need for prospective, randomized clinical studies to genuinely discern the platform’s potential RARP.

Finally, the Versius^®^ surgical robotic system (CMR Surgical, Inc., Cambridge, United Kingdom) has also been explored in RARP. Preliminary reports of its usage involve a pre-clinical study conducted on cadavers [99]. Featuring a modular design, the Versius^®^ system employs dual-console capability, allowing two surgeons to operate independently in different anatomical areas concurrently [100]. To our knowledge, only one RARP involving a living patient has been documented with this platform, executed by Rocco et al. The procedure was completed safely, and the authors drew attention to the Versius model’s distinct features. These include a specialized controller handgrip (comprising a camera and clutch) without pedal control, a handgrip-located energy control attribute, and an instrument length measuring 30 cm [101]. The Versius^®^ surgical robotic system’s efficacy was further validated in a preclinical cadaveric study. This study, adhering to IDEAL-D recommendations, evaluated the system using three-arm and four-arm bedside unit setups. Prostatectomies were successfully completed with both setups, with minor adjustments for surgeon preference. Instrument challenges, particularly with the Monopolar Curved Scissor tip and Needle Holders, were noted and addressed in response to surgeon feedback. Additionally, the system’s versatility was demonstrated by successfully performing cystectomies, suggesting its broader applicability in urological procedures [102]. Further clinical use was explored in a study with 18 patients undergoing RARP for localized prostate cancer, marking a significant step towards clinical application. This study documented median setup, console, operative, and total surgery times, along with the time for bilateral pelvic lymphadenectomy. Notably, no complications or limitations related to the Versius system were reported, and the patients exhibited promising recovery outcomes, including a high rate of continence at two months post-operation. This study underscores the feasibility, safety, and reproducibility of RARP using the Versius system, highlighting its potential for broader clinical adoption [103].

As novel robotic platforms continue to evolve, prospective randomized comparisons across robotic devices should be pursued to help guide surgeons and hospitals as they weigh various options for purchasing and deploying new surgical robots in their practices.

## 8. Conclusions

RARP is constantly evolving in an effort to reduce surgical morbidity and optimize patient outcomes. From the curation of a multitude of diverse technical approaches to the development of entirely new surgical platforms and artificial intelligence tools, several innovations have offered notable benefits for patients. Although new surgical and technological developments warrant continued study, RARP serves as an incubator for surgical innovation, and we are excited to witness continued progress in the surgical treatment of men with prostate cancer.

## Figures and Tables

**Table 1:** Pelvic-fascia-sparing techniques in the literature.

Study Authors	Chang et al. [66]	Umari et al. [60]	Egan et al. [61]	Menon et al. [62]	Abdel Raheem [63]	Qiu et al. [64]
**Approach**	Retzius sparing	Retzius sparing	Retzius sparing	Retzius sparing	Retzius sparing	Retzius sparing
**Study design**	Retrospective (*n* = 30)	Prospective (*n* = 282)	Prospective (*n* = 70)	Randomized Control Trial (*n* = 60)	Prospective (*n* = 125)	Randomized Control Trial (*n* = 55)
**Age (yr),** **median** **(IQR)**	64 +/− 7	63 (57–69)	62 (55–69)	61.0 (55–67)	62 (56.5–67.5)	68 (62–71)
**BMI** **(kg/m^2^),** **median** **(IQR)**	na	26 (21–31)	28.4 (23.7–33.1)	27.9 (26.1–30.6)	27.9 (26.1–30.6)	24.2 (22.3–26.4)
**PSA (ng/mL),** **median** **(IQR)**	18 +/− 19	6.4 (4.6–9.1)	7.2 (4–10.4)	5.7 (4.7–7.4)	7.0	9.1 (5.6–15.1)
**Biopsy** **Gleason** **Score (%)**	na	GS 6: 15.6 GS 7: 69.1 GS 8+: 13.1	na	GS 6: 30 GS 7: 70	GS 6: 31 GS 7: 50 GS 8+: 19	GS 6: 43.6 GS 7: 38.1 GS 8+: 18.2
**Clinical T** **stage (%)**	na	T1: 37.2 T2: 42.9 T3: 19.9	T1: 71.4 T2: 18.6 T3: 10	T1: 67 T2: 32 T3: 1.7	na	T1c: 12.7 T2a–T2b: 70.9 T2c: 16.4
**Risk** **Category** **(%)**	na	D’Amico Low: 11.3 Int: 51.4 High: 37.2	na	NCCN Low: 23 Int: 77	na	D’Amico Low/intermediate: 65.5 High: 34.5
**IPSS (IQR)**	na	na	na	7 (3–12)	na	1 (0–4)
**Sexual Health Inventory for Men (SHIM) (IQR)**	na	na	na	20 (14–24)	na	na
**International Index of Erectile Function—5 (IIEF)**	na	Baseline: 19.4 1 mo: 7.6 6 mo: 8.1 12 mo: 8.9	na	na	na	na
**Operative time (min),** **median (IQR)**	211.83 (168.86–254.8)	150 (120–170)	na	160 (141–180)	na	105 (85–125)
**Console time (min), median (IQR)**	na	110 (90–138)	130 +/− 26.1 mean +/− SD	115 (98–130)	na	na
**Length of stay (day), median (IQR)**	na	2 (2–2)	1.1 +/− 0.4 mean +/− SD	1 (1–1)	na	na
**Blood loss (mL),** **median (IQR)**	150 +/− 109	79 (28)	100 (75–200)	75 (50–100)	225 (162–288)	200 (200–300)
**Transfusion (%)**	na	5 (1.8)	na	na	na	na
**Complications, Clavien–Dindo grade (%)**	na	Clavien–Dindo 1: 3 2: 7 3: 5 4–5: 0	na	na	na	I: 5.5 II: 0 III: 0 IV–V: 0
**Pathologic** **stage (%)**	pT2: 16 pT3: 14	pT2: 70.6 pT3: 29.4	pT2: 67.1 pT3a: 20 pT3b: 12.9	≤pT2: 55 pT3a: 35 pT3b: 10	pT2: 74 pT3: 22 pT4: 4	na
**Positive Surgical Margin (%)**	0.2	15.6	34.3	Organic confined disease (≤pT2c): 15.2 Extra-prostatic disease (≥pT3a): 37	25.0	23.6
**Early continence**	1 week: 73% 1 month: 91% 3 months: 94% 6 months: 98%	Immediate: 70.4%	na	Immediate: 60% 3 months: 74% 6 months: 88%	3 months: 73% 6 months: 82% 12 months: 90%	Immediate: 69% 6 months: 88% 12 months: 93%
**Study Authors**	**Asimakopoulos et al. [65]**	**Sayyid et al. [67]**	**Wagaskcar et al. [70]**	**Shimmura et al. [71]**	**Zhang et al. [72]**	
**Approach**	Retzius sparing	Retzius sparing	Hood sparing	Hood sparing	Hood sparing	
**Study design**	Randomized Control Trial (*n* = 39)	Prospective (*n* = 100)	Prospective (*n* = 300)	Retrospective (*n* = 42)	Retrospective (*n* = 24)	
**Age (yr),** **median** **(IQR)**	66 (61–71)	61.0 (57.0–66.0)	64 (58–68)	74.0 (70.0–80.0)	70.0 (64.5–76.5)	
**BMI** **(kg/m^2^),** **median** **(IQR)**	na	29.0 (26.0–32.0)	27 (25–29)	23.9 (21.9–26.7)	24.9 (22.7–26.8)	
**PSA (ng/mL),** **median** **(IQR)**	7 (5.5–8.3)	8.8 (6.4–12.0)	6 (4–8)	9.2 (5.3–14.0)	17.0 (3.5–36.4)	
**Biopsy** **Gleason** **Score (%)**	GS 6: 69.2 GS 7: 30.7	GS 6: 19 GS 7: 62 GS 8+: 19	GS 6: 16 GS 7: 65 GS ≥8: 19	GS 6: 5 GS 7: 62 GS ≥8: 33	GS 6: 13 GS 7: 54 GS ≥8: 33	
**Clinical T** **stage (%)**	T1c: 77 T2a–T2b: 20.5 T2c: 2.5	≤T2: 73 ≥T3: 27	T1: 51 T2: 35 T3: 14	T1: 21 T2: 76 T3: 3	T1: 25 T2: 37 T3: 38	
**Risk** **Category** **(%)**	na	D’Amico Low: 24 Int: 49 High: 27	CAPRA Low: 12 Int: 66 High: 22	na	D’Amico Low: 12 Int: 50 High: 38	
**IPSS (IQR)**	na	9.0 (5.0–13.0)	8 (4–14)	na	15 (13–22)	
**Sexual Health Inventory for Men (SHIM) (IQR)**	na	na	na	na	na	
**International Index of Erectile Function—5 (IIEF)**	na	na	57 (35–67)	na	na	
**Operative time (min),** **median (IQR)**	179.8 (138.9–220.7)	na	169 (147–195)	151.5 (131.0–168.0)	182.5 (141.0–208.3)	
**Console time (min), median (IQR)**	na	120.0 (105.0–142.0)	118.5 (100–141)	121.5 (103.0–141.0)	na	
**Length of stay (day), median (IQR)**	na	1.0 (1.0–1.0)	na	na	na	
**Blood loss (mL),** **median (IQR)**	na	100.0 (50.0–200.0)	150.0	79.0 (50.0–135.0)	170.0 (25.0–300.0)	
**Transfusion (%)**	na	0.0	0.0	na	0.0	
**Complications, Clavien–Dindo grade (%)**	na	na	I: 2.3 II: 5.7 III: 1.7	na	I–II: 12.5 III–V: 0	
**Pathologic** **stage (%)**	pT2: 53.8 pT3a: 35.9 pT3b: 7.7	≤pT2: 66 ≥pT3: 34	pT2: 81 pT3: 19	pT2: 67 pT3: 24	pT2: 38 pT3: 62	
**Positive Surgical Margin (%)**	28.9	27.0	6.0	16.0	8.0	
**Early continence**	Immediate: 51% 1 month: 81% 3 months: 90.5% 6 months: 90.5%	3 months: 59% 12 months: 97%	1 week: 21% 1 month: 69% 3 months: 91%	2 weeks: 36% 1 month: 69% 3 months: 91% 6 months: 100%	1 week: 54% 1 month: 75% 3 months: 92%	

na: not applicable.

## Data Availability

Not applicable.

## Abbreviations

BR	Biochemical recurrence
IIEF-5	International Index of Erectile Function—5
PSM	Positive surgical margins
RARP	Robotic-assisted radical prostatectomy
SHIM	Sexual Health Inventory for Men
SP	Single Port
